# Midkine-a Protein Localization in the Developing and Adult Retina of the Zebrafish and Its Function During Photoreceptor Regeneration

**DOI:** 10.1371/journal.pone.0121789

**Published:** 2015-03-24

**Authors:** Esther Gramage, Travis D’Cruz, Scott Taylor, Ryan Thummel, Peter F. Hitchcock

**Affiliations:** 1 Department of Ophthalmology and Visual Sciences, University of Michigan, Ann Arbor, Michigan, United States of America; 2 Departments of Anatomy and Cell Biology and Ophthalmology, Wayne State University School of Medicine, Detroit, Michigan, United States of America; University of Notre Dame, UNITED STATES

## Abstract

Midkine is a heparin binding growth factor with important functions in neuronal development and survival, but little is known about its function in the retina. Previous studies show that in the developing zebrafish, Midkine-a (Mdka) regulates cell cycle kinetics in retinal progenitors, and following injury to the adult zebrafish retina, *mdka* is strongly upregulated in Müller glia and the injury-induced photoreceptor progenitors. Here we provide the first data describing Mdka protein localization during different stages of retinal development and during the regeneration of photoreceptors in adults. We also experimentally test the role of Mdka during photoreceptor regeneration. The immuno-localization of Mdka reflects the complex spatiotemporal pattern of gene expression and also reveals the apparent secretion and extracellular trafficking of this protein. During embryonic retinal development the Mdka antibodies label all mitotically active cells, but at the onset of neuronal differentiation, immunostaining is also localized to the nascent inner plexiform layer. Starting at five days post fertilization through the juvenile stage, Mdka immunostaining labels the cytoplasm of horizontal cells and the overlying somata of rod photoreceptors. Double immunolabeling shows that in adult horizontal cells, Mdka co-localizes with markers of the Golgi complex. Together, these data are interpreted to show that Mdka is synthesized in horizontal cells and secreted into the outer nuclear layer. In adults, Mdka is also present in the end feet of Müller glia. Similar to *mdka* gene expression, Mdka in horizontal cells is regulated by circadian rhythms. After the light-induced death of photoreceptors, Mdka immuonolabeling is localized to Müller glia, the intrinsic stem cells of the zebrafish retina, and proliferating photoreceptor progenitors. Knockdown of Mdka during photoreceptor regeneration results in less proliferation and diminished regeneration of rod photoreceptors. These data suggest that during photoreceptor regeneration Mdka regulates aspects of injury-induced cell proliferation.

## Introduction

Midkine is a heparin-binding growth factor that forms a two-member family with Pleiotrophin. Both factors are abundantly expressed during embryogenesis, with particularly high levels in the developing nervous system [1]. Beyond mid-gestation and during postnatal stages, the expression of *midkine* and *pleiotrophin* are rapidly downregulated [2–6]. Genes encoding both Midkine and Pleiotrophin are up-regulated under disease conditions, most notably those that affect the nervous system [7–11]. For example, in rodents, Midkine is upregulated after retinal damage [11], and the up-regulation of *midkine* and *pleiotrophin* coincides with cytokine activity during nervous system repair [12–15]. Throughout the nervous system Midkine is proposed to play a role in reparative mechanisms.

The retina is used extensively as a model to study brain development, injury and diseases [16]. It is comprised of a precisely patterned arrangement of six neuronal classes, that include two classes of photoreceptors (rods and cones), three classes of interneurons (horizontal cells, bipolar cells and amacrine cells), ganglion cells that serve as the output neurons and one glial cell type (Müller glia). Although both structure and function of the retina are highly conserved among vertebrates, there are vast differences between species in their ability to regenerate neurons following injury [17]. Mammals have an almost nonexistent capacity for neuronal regeneration. In stark contrast, any lesion that kills retinal neurons in zebrafish leads to complete neuronal regeneration [18–22]. This robust regenerative neurogenesis in the retina is dependent on Müller glia, which act as intrinsic stem cells and give rise to progenitors capable of replenishing each of the six neuronal cell types.

Several paradigms have been used to study neuronal regeneration in the zebrafish retina [23–29]. A photolytic lesion, which results in the selective death of photoreceptors [25,30,31], is widely used and serves as a model of human photoreceptor dystrophies [32]. The death of the photoreceptors stimulates Müller glia to re-enter the cell cycle, divide and give rise to rapidly proliferating progenitors that form radial clusters surrounding the parent Müller glia. These progenitors then migrate to ONL, exit the cell cycle and differentiate into both rod and cone photoreceptors (reviewed in [33]). Cones are regenerated prior to rods, and the first regenerated cones begin to appear at around 4 days post-lesion [34]. The depleted ONL is functionally reconstituted within 15–20 days [35].

The mechanisms leading to the de-differentiation of Müller glia and has been intensively studied. For example, dying photoreceptors signal to Müller glia by synthesizing and secreting TNF-α [36]. Several transcription factors and signaling pathways are then required for Müller glia to re-enter the cell cycle and to sustain proliferation, including Ascl1a [37], Insm1a [37], Stat3 [36], FGF [38], TGF-β [21] and Stil [39].

In zebrafish, there are two *midkine* paralogs, *midkine-a* (*mdka*) and *midkine-b* (*mdkb*), which share 68% of amino acid identity [40]. Both midkines are differentially regulated during brain development [40], and both are upregulated in zebrafish during regeneration of multiple tissues and organs, e.g., heart [41], fin [42] and retina [10]. *mdka* and *mdkb* were first identified in the retina by an unbiased screen for genes induced by the death of the photoreceptors and during photoreceptor regeneration [43]. The same study showed that during embryonic retinal development, *mdka* and *mdkb* have distinct cellular patterns of expression. A subsequent study of Mdka function showed that in retinal progenitors this protein governs cell cycle kinetics. Loss- and gain-of-function slows and accelerates the cell cycle, respectively [44].

The goal of the present study was to establish the patterns of Mdka protein localization during retinal development and photoreceptor regeneration and gain insight into its extracellular trafficking and function during these events. The expression of *mdka* in Müller glia and photoreceptor progenitors after light-induced lesion [43] and the known roles of Midkine in neural repair, lead us to experimentally test the hypothesis that Mdka has a fundamental function in governing Müller glial-based photoreceptor regeneration. The data show that during embryonic retinal development the antibodies against Mdka labels all mitotically-active cells. As progenitors exit the cell cycle and begin to differentiate, Mdka immunostaining becomes restricted to horizontal cells. Coinciding with this transition, the Mdka antibodies transiently label the inner plexiform layer, and the immunostaining of photoreceptors in the outer nuclear layer first appears. In the adult retina, the Mdka antibodies label horizontal cells, rod photoreceptors and the end feet of Müller glia. For horizontal cells, Mdka immunostaining is present in the cytosol, co-localizes with markers of the Golgi complex and is strongly regulated by the circadian rhythm. During photoreceptor regeneration, the Mdka antibodies label Müller glia and retinal progenitors. Following knockdown of Mdka, there are fewer retinal progenitors and regenerated rods, indicating that Mdka governs proliferation of injury-induced progenitors.

## Methods

### Animals and retinal lesions

AB strain zebrafish (*Danio rerio*) were purchased from the Zebrafish International Research Center (ZIRC, University of Oregon, Eugene, Oregon), propagated in-house and housed in recirculating systems at 28.5°C on a 14/10-h light/dark cycle. For the development studies, embryos were collected within 15 minutes of spawning and incubated at 28.5°C on a 14/10-h light/dark cycle. All experimental protocols were approved by the University of Michigan’s committee for the Use and Care of Animals.

### Knockdown of Mdka in embryos

Morpholino oligonucleotides (MOs; Gene Tools, LLC, Cowallis, OR, USA) were used to knockdown Mdka in embryos. Previously described and validated protocols for the ATG-targeted *mdka* MOs were used (see [44]). Embryos were injected at the 1-cell stage with 3ng of Mdka MO (5’-CCGCATTTTGTTTTCTGTGTCGAAA-3’) or mismatch control (Mdka MM, 5’-CCGgATTTTcTTTTCTcTcTgGAAA-3’) diluted in 1x Danieau buffer [45], and analyzed at 48 hours post fertilization (hpf).

### Systemic labeling with BrdU or EdU

Proliferating cells were labeled in adult fish with either 5-Bromo-2´-deoxyuridine (BrdU) or 5-ethynyl-2’-deoxyuridine (EdU) by swimming the fish for 24 hours in 5 mM BrdU or 250 μM EdU solution [46,47]. For BrdU staining, sections were incubated in 100°C sodium citrate buffer (10 mM sodium citrate, 0.05% Tween 20, pH 6.0) for 30 minutes to denature DNA and cooled at room temperature for 20 minutes. Sections were then subjected to standard immunolabeling as described below. EdU was visualized using the Click-it EdU kit (Invitrogen, Carlsbad, CA, USA).

### Transient transgenesis

To visualize the horizontal cells in the retina, a DNA construct containing the Connexin 55.5 promoter driving expression of Enhanced Green Fluorescent Protein (Cx55.5:EGFP) in a Tol2 destination vector (a gift from Prof. Maarten Kamermans; [48]) was used to create a transient transgenic zebrafish. The DNA construct was co-injected with Tol2 transposon mRNA in 1-cell-stage wild type embryos. EGFP immunolabeling was performed as described below.

### Immunohistochemistry

Whole zebrafish (30hpf to 2 months of age) or eye cups (from adults) were fixed by immersion in buffered 4% paraformaldehyde, cryoprotected by infiltration in 20% sucrose in phosphate buffer, and frozen in Optimal Cutting Temperature compound (OCT; e.g., [44]). Ten micron cryosections were mounted on glass slides and processed for immunohistochemistry or *in situ* hybridization combined with immunohistochemistry.

For immunostaining, sections were incubated in PBS at 37°C for 15 min, washed with PBS containing 0.5% Triton X-100 (PBST), and incubated with a blocking solution containing 5% BSA and 10% sheep serum in 20mM MgCl_2_ PBS. This was followed by overnight incubation at 4°C with primary antibodies. The next day, sections were washed with PBST and incubated in secondary antibodies for 1 hour at room temperature, washed again with PBST, counterstained with 1:1000 dilution of Hoechst to label nuclei and sealed with mounting media and glass coverslips.

The primary antibodies used were: rabbit anti-Mdka 1:200 (Zymed Laboratories-Invitrogen; see [49]), mouse anti-BrdU 1:100 (BD Biosciences 347580), mouse anti-GM130 1:200 (BD Biosciences 610822; [50]), mouse anti-Glutamine Synthetase 1:200 (Millipore MAB302), mouse anti-GFP 1:200 (Millipore MAB3580), mouse anti-Zpr1 1:200 (anti-Arrestin 3, ZIRC), mouse anti-Zpr3 1:200 (anti-Rhodopsin, ZIRC). Secondary antibodies (Invitrogen) were raised against in either mouse or rabbit antibodies and conjugated to Alexa Fluor 488 and 555 and diluted 1:500. Fluorescence images were captured using a Leica TCS SP5 confocal microscope (Vernon Hills, IL, USA).

### *In situ* hybridization

*In situ* hybridizations on retinal sections were performed using previously published protocols (e.g., [44,51]). Briefly, sections were hybridized with digoxigenin (DIG)-labeled riboprobes *rhodopsin (rho)* for rod photoreceptors or *phosphodiesterase 6c (pde6c)* for cone photoreceptors overnight at 55°C, incubated with an alkaline-phosphatase-conjugated anti-DIG antibody overnight at 4°C and visualized using 4-nitrobluetetrazolium/5-bromo-4-chloro-3-indolyl phosphate (NBT/BCIP) as a substrate. When *in situ* hybridizations were combined with BrdU immunolabeling, sections were post-fixed in buffered 4% paraformaldehyde for 10 minutes and rinsed for 1 hour in several changes of PBS. Sections were then immunolabeled for BrdU as described above. Bright field and fluorescence images in double-labeled sections were captured with a Leica DM6000 microscope (Vernon Hills, IL, USA).

### Western Blot Analysis

Protein samples were obtained by pooling 50 embryo heads in a lysis buffer with protease inhibitors (Complete Mini, Roche, Germany). Proteins were separated in a 12% SDS-PAGE gel and transferred to a nitrocellulose membrane (Sigma, St. Louis, MO, USA). The membrane was blocked in 5% non-fat dry milk in PBS for 2 hours and incubated with rabbit anti-Mdka antibodies (1:1,000) (Zymed Laboratories-Invitrogen, Carlsbad, CA, USA). Blots were rinsed with PBS and incubated with goat horseradish peroxidase- conjugated secondary IgG (1:5,000) for 1 hour. Bound antibodies were visualized using the enhanced chemiluminescence assay (ECL detection system, Amersham Biosciences, Arlington Heights, IL, USA). As loading controls, blots were also incubated with anti-actin (1:1,000, Calbiochem, Germany). Images were captured using the FluorChem E Imaging System (Bio-Techne Minneapolis, MN, USA).

### Photolytic lesions, morpholino electroporation and cell counts

To deplete photoreceptors, adult pigmented fish were housed in complete darkness for 24 hours, then immediately exposed to high intensity light (ca. 100,000 lux) from a mercury arc lamp for 30 minutes [25,52]. Fish were then moved to a light box and exposed to constant light (ca. 30.000 lux) for 72 hours [52]. All photolytic lesions were started at the same time of day to avoid any potential influence from circadian rhythms (see S1 Fig.). Days post lesion (dpl) indicate the number days following the onset of the constant light exposure.

At 1 dpl, lissamine-tagged antisense Mdka MOs (see above) were injected (3 mM; 0.5 μl) into the vitreous chamber of one eye and electroporated into dorsal retina using methods and settings described previously [53]. Electroporation of standard control MOs (Gene Tools, LLC) served as the control. At 2 dpl, all animals were housed overnight in a solution containing 5mM BrdU to label proliferating cells. Animals were then sacrificed either at 3 dpl or returned to normal intensity and cyclical lighting and sacrificed at 6 dpl. All animals were sacrificed at the same time of day to avoid the influence of the circadian rhythms. Eyes were excluded from any further analysis if they showed damage from the electroporation, contained incomplete lesion or there was persistence of Mdka immunofluorescence. Two and three independent experiments were performed for animals at 3 and 6 dpl, respectively. For each eye, the average cell count from 3 non-adjacent cross sections spanning 500μm of the central retina was calculated. This value was averaged across 4–7 eyes from each treatment group to obtain a single value.

To count BrdU-labeled cells from retinas at 3 dpl, a confocal z-stack was collected from three non-adjacent sections through the central retina of each eye using a Leica SP5 confocal microscope (Vernon Hills, IL, USA). The number of BrdU-positive cells was counted in a 500 μm length of retina from within the lesion using Imaris Software (Bitplane, South Windsor, CT, USA), using standardized parameters for sensitivity and cell size.

Sections from the 6 dpl group were processed for *in situ* hybridization for either *rhodopsin* (rods photoreceptors) or *pde6c* (cone photoreceptors) and BrdU immunostaining. BrdU-labeled (regenerated) rods or cones were then counted manually in a 500 μm length of retina from within the lesion.

### Statistical Analysis

Statistical significance of cell counts between control and experimental retinas was determined with a Student’s t-test using GraphPad Prism 5 (La Jolla, CA, USA). A p-value less than 0.05 was considered a statistically significant difference.

## Results

### Cellular localization of Mdka during retinal development and in the adult retina

To test the specificity of the Mdka antibodies, detection of Mdka and its absence in *mdka* morphants was confirmed using Western blots (see also [44]), Then the antibodies were used for immunofluorescence on retinal cryosections taken from control and morphant embryos. Western blots (S2A Fig.) show that the Mdka antibodies recognize a major band at around 13 kDa, and this band is absent in embryos injected with the *mdka* MOs. The source of the minor band (≈16 kDa) in the Western blot is not clear. However, the presence of this band after Mdka knockdown (S2B Fig.) suggests that the band is likely a protein that is not Mdka. In tissue sections from 48hpf embryos (S2B Fig.), the *mdka* morphants show the microphthalmia characteristic of Mdka knockdown [44] and a complete absence of the immunofluorescence signal. This is in marked contrast to the immunostaining for uninjected and control embryos (taken from the same clutch), which show the pattern of immunostaining characteristic of the 48hpf retina (see below). These data demonstrate that the polyclonal antibodies used here to characterize protein localization in retinal sections are specific for Mdka.

**Fig 1:**
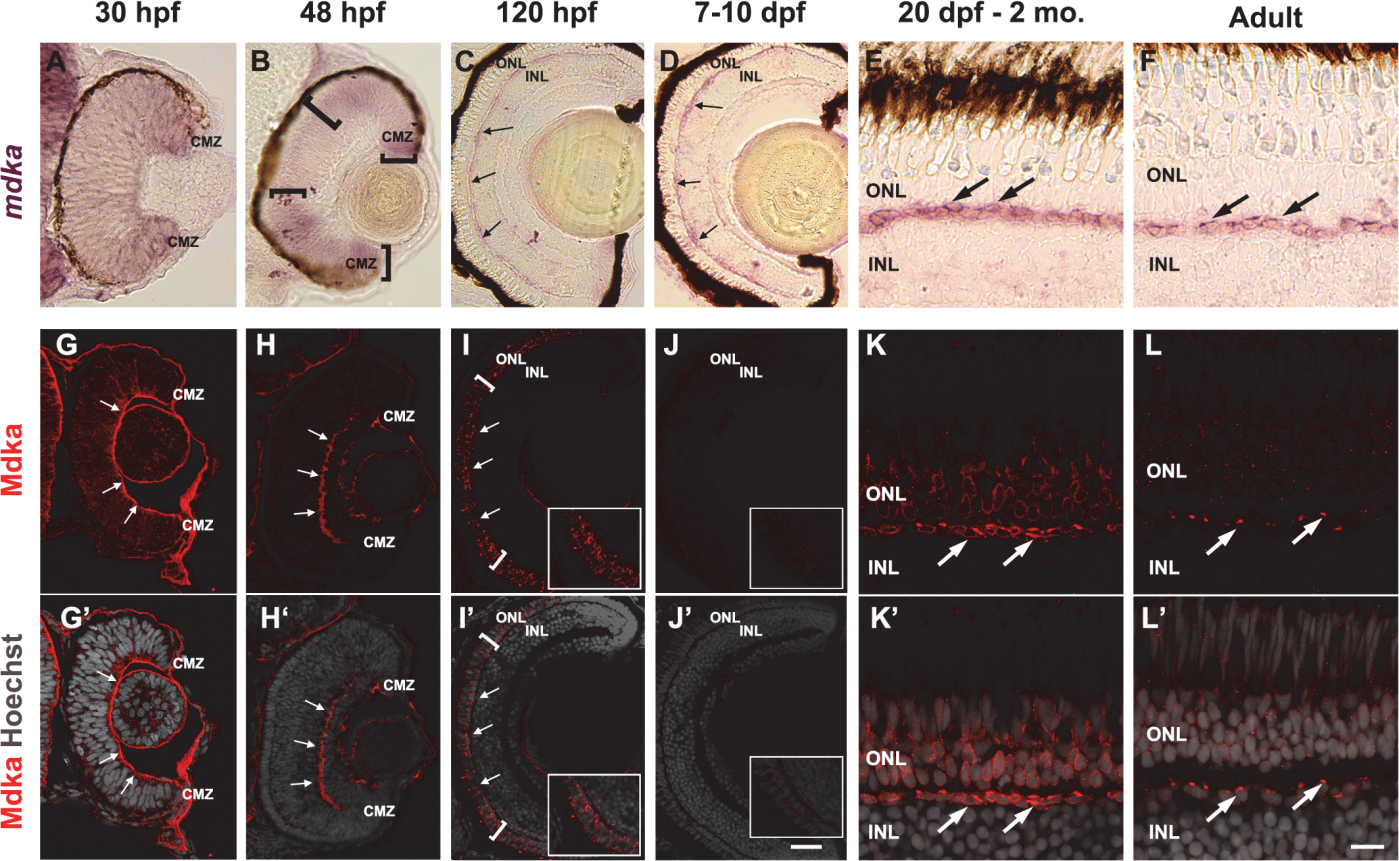
*mdka* expression and Mdka immunostaining during retinal development and in adults. *In situ* hybridization showing *mdka* expression at stages between 30hpf and adults (A-F). Mdka immunostaining at stages corresponding to the *in situ* hybridizations are also illustrated (G-H and images merged DAPI counterstain below). At 30 hpf, Mdka immunofluorescence in strongest at the basal surface of the retina (panel G, arrows). At 48hpf, Mdka antibodies transiently label the nascent inner plexiform layer (panel H, arrows). At 120hpf, when differentiated photoreceptors are present, Mdka immunostaining is present in horizontal cells (arrows) and the outer nuclear layer (brackets). Though tested numerous times, the level of Mdka immunofluorescence dramatically decreases between 7–10 dpf (panel J, J’). Note that at this time point horizontal cells express *mdka* (panel D). In the juvenile retina, Mdka immunostaining returns and both horizontal cells (arrows, panel K, K’) and the overlying rod photoreceptors in the ONL are intensely stained. This juvenile pattern of immunostaining is present in adults, but the overall intensity of the immunostaining is reduced, and the immunostaining of horizontal cells is consolidated to a small immunofluorescent plaque lying adjacent to each DAPI-stained nucleus (arrows, panel L’). hpf, hours post fertilizations; dpf, days post fertilization; mo, months; ONL, outer nuclear layer; INL, inner nuclear layer; IPL, inner plexiform layer; CMZ, circumferential marginal zone. Scale bar in panel J’ equals 20 μm and corresponds to images in columns A-D and G-J. Scale bar in panel L’ equals 10 μm and corresponds to images in panels E, F, K, K’, L and L’.

Next, to gain insights into the cellular localization of Mdka synthesis and potential location of the secreted protein, comparisons were made between *in situ* hybridizations using *mdka* probes and Mdka immunostaining. The cellular pattern of *mdka* expression changes markedly throughout development in the zebrafish retina ([43]; Fig. 1A-F). At 30 hpf (Fig. 1A), prior to significant neuronal differentiation, *mdka* is expressed throughout the neuroepithelium, with higher expression at the retinal margin, presaging the circumferential marginal zone (CMZ) of the mature retina [54]. At 48 hpf (Fig. 1B), as progenitors withdraw from the cell cycle and differentiation commences centrally, *mdka* is down-regulated in newly postmitotic cells, while expression persists in the still dividing cells in the periphery. The center-to-periphery pattern of neuronal differentiation continues, such that by 120 hpf (Fig. 1C), when the retina is fully differentiated, *mdka* expression is restricted to the CMZ and horizontal cells (see also [43]). This cellular pattern of expression remains invariant during subsequent growth and into adulthood (Fig. 1D-F).

Similar to the developmental pattern of *mdka* expression, the developmental pattern of Mdka immunolabeling is also temporally and spatially dynamic. At 30 hpf (Fig. 1G, G’), the Mdka immunolabel is present throughout the retinal neuroepithelium, though the immunostaining is concentrated at the basal surface. At 48hpf (Fig. 1H, H’), there is an apparent reduction of Mdka immunostaining of dividing cells and at the basal surface, and the Mdka antibodies transiently labels the nascent inner plexiform layer. At 120 hpf (Fig. 1I, I’), reflecting the cellular pattern of gene expression, Mdka immunostaining is present in horizontal cells (arrows). However, in contrast to the *mdka* expression, which is restricted to horizontal cells, at 120hpf, Mdka antibodies also label photoreceptors (brackets). Unexpectedly, though repeatedly tested, between 7–10 days post fertilization (Fig. 1J, J’), Mdka immunostaining is undetectable in retinal sections. This transient absence of immunostaining is restored by 20 days post fertilization (Fig. 1K, K’), when high levels of Mdka immunostaining is present both among horizontal cells and the overlying outer nuclear layer, where the immunostaining is restricted to rod photoreceptors (S3 Fig.). In the adult retina (Fig. 1L, L’), the overall levels of Mdka immunostaining are diminished, and Mdka immunostaining in horizontal cells appears as a small plaque of fluorescence on the apical aspect of each nucleus. The intensity of the Mdka immunofluorescence surrounding each rod photoreceptor is noticeably diminished in the adult retina as well when compared to juveniles.

**Fig 2:**
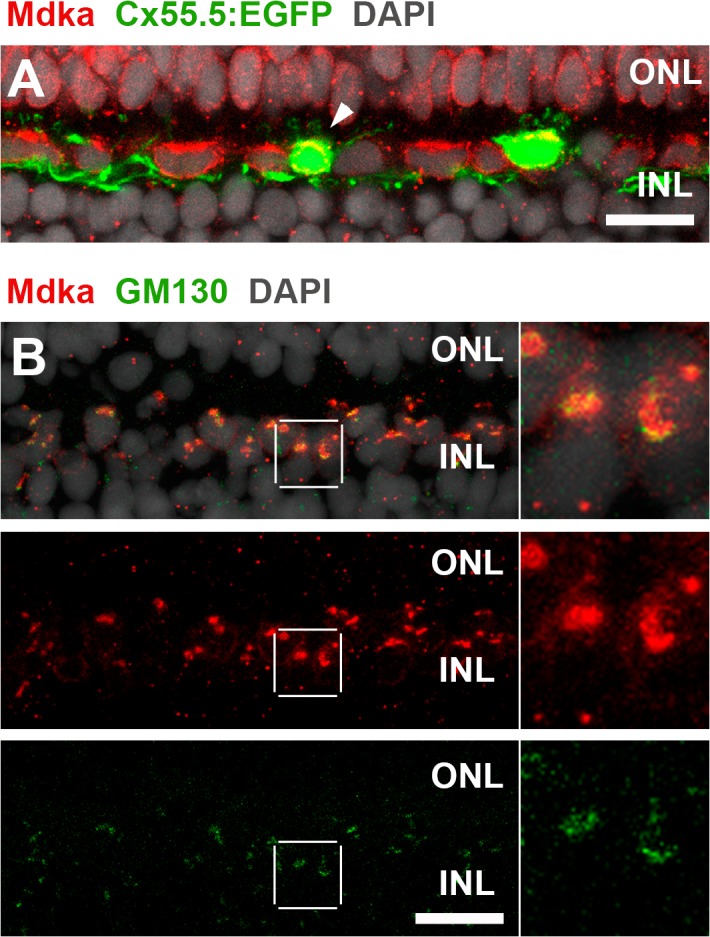
Mdka immunostaining of horizontal cells is intracellular and colocalizes with markers of the Golgi complex. In fish injected with the Cx55.5:EGFP construct, immunolabels for both Mdka and EGFP colocalize to the cytoplasm of horizontal cells (arrowhead, panel A). In the adult retina, Mdka immunostaining colocalizes with the antibody marker of the Golgi complex (column B; and insets). ONL: outer nuclear layer; INL: inner nuclear layer. Scale bars equal 10 μm.

The immunostaining alone of the horizontal cells was insufficient to establish if the Mdka immunolabel was localized to an intracellular compartment or concentrated on the cell surface. To address this, we generated transient transgenic animals that expressed enhanced green fluorescent protein (EGFP) under the control of the promoter of *connexin 55*.*5*, a gene expressed exclusively by horizontal cells in the teleost retina [48]. Retinal sections from transgenic animals were immunostained for both EGFP, which diffusely fills the cytoplasm, and Mdka. These experiments show co-localization of the GFP and Mdka immunofluorescence, demonstrating that that in horizontal cells the Mdka immunolabel is cytosolic (Fig. 2A). Based on the size, shape and proximity to the nucleus of the intracellular plaque of Mdka immunostaining in the adult retina, we hypothesized that the Mdka immunostaining colocalizes with the Golgi complex. Double immunostaining for Mdka and the Golgi marker, GM130, showed that in each horizontal cell, the Mdka immunostaining colocalizes with markers of the Golgi complex (Fig. 2B).

**Fig 3:**
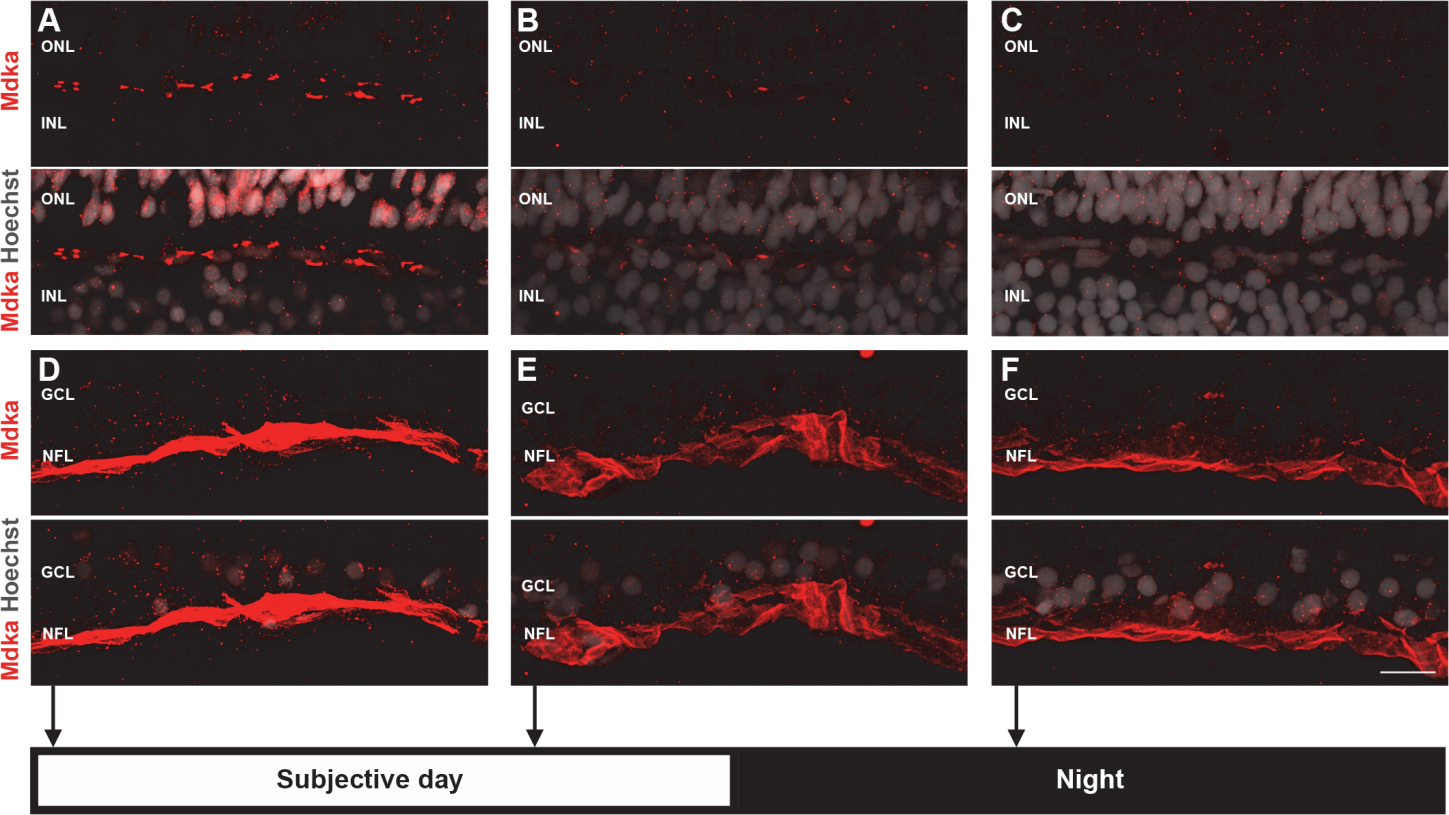
Circadian regulation of Mdka immunostaining in horizontal cells. At the onset of the subjective day, Mdka immunostaining of horizontal cells is maximum (column A), decreases throughout the subjective day (column B) and reaches a minimum during the subjective night (column C). In contrast, there is no corresponding substantial change in the intensity of immunostaining within the endfeet of the Müller glia (D-F). ONL: outer nuclear layer; INL: inner nuclear layer; GCL: ganglion cell layer; NFL: nerve fiber layer. Scale bar = 10 μm.

**Fig 4:**
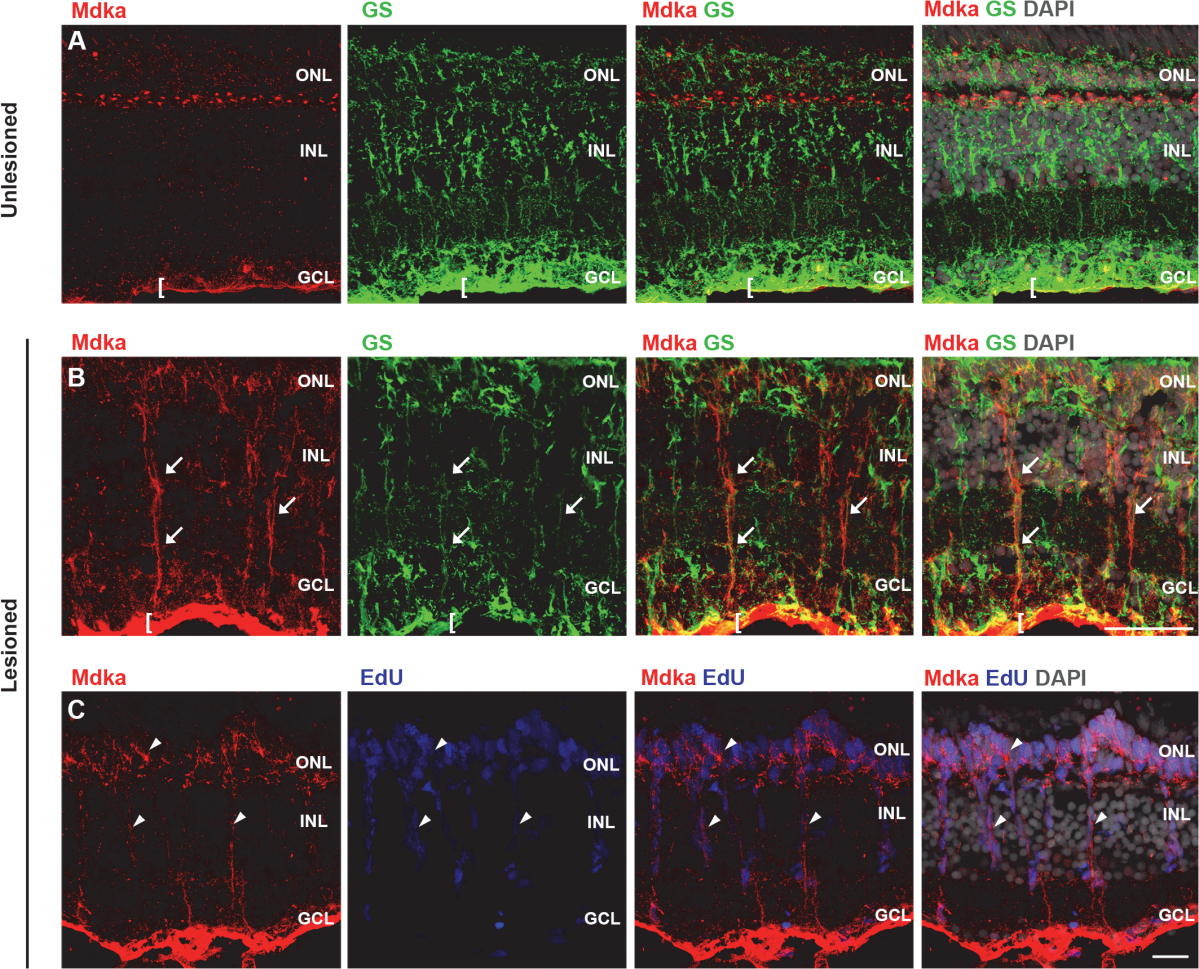
Mdka protein localization following photoreceptor ablation. In unlesioned retinas, Mdka immunostaining is localized to the horizontal cells and endfeet of glutamine synthetase (GS)-positive Müller glia (row A). At 4 dpl, Mdka antibodies label the radial processes of Müller glia (row B, arrows). Note the increased Mdka immunostaining in the endfeet of the Müller glia in lesioned retinas (cf. rows A and B). Also at 4 dpl, Mdka immunostaining is localized to each of the EdU-positive nuclei in both the INL and ONL (row C, arrowheads). ONL: outer nuclear layer; INL: inner nuclear layer; dpl: days post lesion. Scale bars = 25 μm.

Finally, also in the adult retina, Mdka protein is present in the end feet of the Müller glia (Fig. 3D-F; S4 Fig.). Double immunostaining for Mdka and glutamine synthetase (GS), which is specific for Müller glia [23,55,56], shows that the thin ribbon of Mdka immunostaining at the basal surface of the retina colocalizes with GS immunostaining that marks the end feet of Müller glia (Fig. 4A). Further, there was no systematic change in the thickness of this label, from retinal periphery to the optic nerve head, allowing us to conclude that the antibodies do not stain ganglion cell axons in the optic fiber layer (data not shown).

### Mdka synthesis is regulated by circadian rhythms

It was shown previously that in the retina of adult zebrafish, *mdka* expression is strongly regulated by the circadian clock. *mdka* levels are highest early in the subjective day, then decrease during the day and into the initial hours of the subjective night [49]. To determine if Mdka protein synthesis and/or its putative secretion are regulated similarly, we repeated the experimental rearing conditions used previously and immunostained retinal sections with the Mdka antibodies at various points during the circadian cycle. This experiment showed that Mdka immunostaining follows a circadian pattern that reflects the mRNA (Fig. 3A-C). Mdka immunofluorescence in horizontal cells and the overlying rod photoreceptors is highest at the onset of the subjective day and decreases into the early hours of the subjective night. In contrast to this circadian pattern, Mdka protein levels remain unchanged (Fig. 3D-F).

### Mdka regulates injury-induced cell proliferation and the regeneration of rod photoreceptors

After a photolytic lesion, *mdka* is expressed in Müller glia and the injury-induced photoreceptor progenitors, suggesting Mdka has a functional role during photoreceptor regeneration [43]. As a prelude to testing the function of Mdka, we established the cellular pattern of Mdka immunostaining in the retinas of animals at 4 dpl, which corresponds to the peak of the injury-induced cell proliferation following the photolytic death of photoreceptors [57]. At this time point, Mdka immunostaining is distributed along the radial processes of the Müller glia throughout the thickness of the retina. Particularly dense Mdka immunostaining is present in the glial endfeet at the basal surface of the retina (Fig. 4B). Mdka immunolabel also surrounds each proliferative, EdU-positive nucleus in the ONL and INL (Fig. 4C).

**Fig 5:**
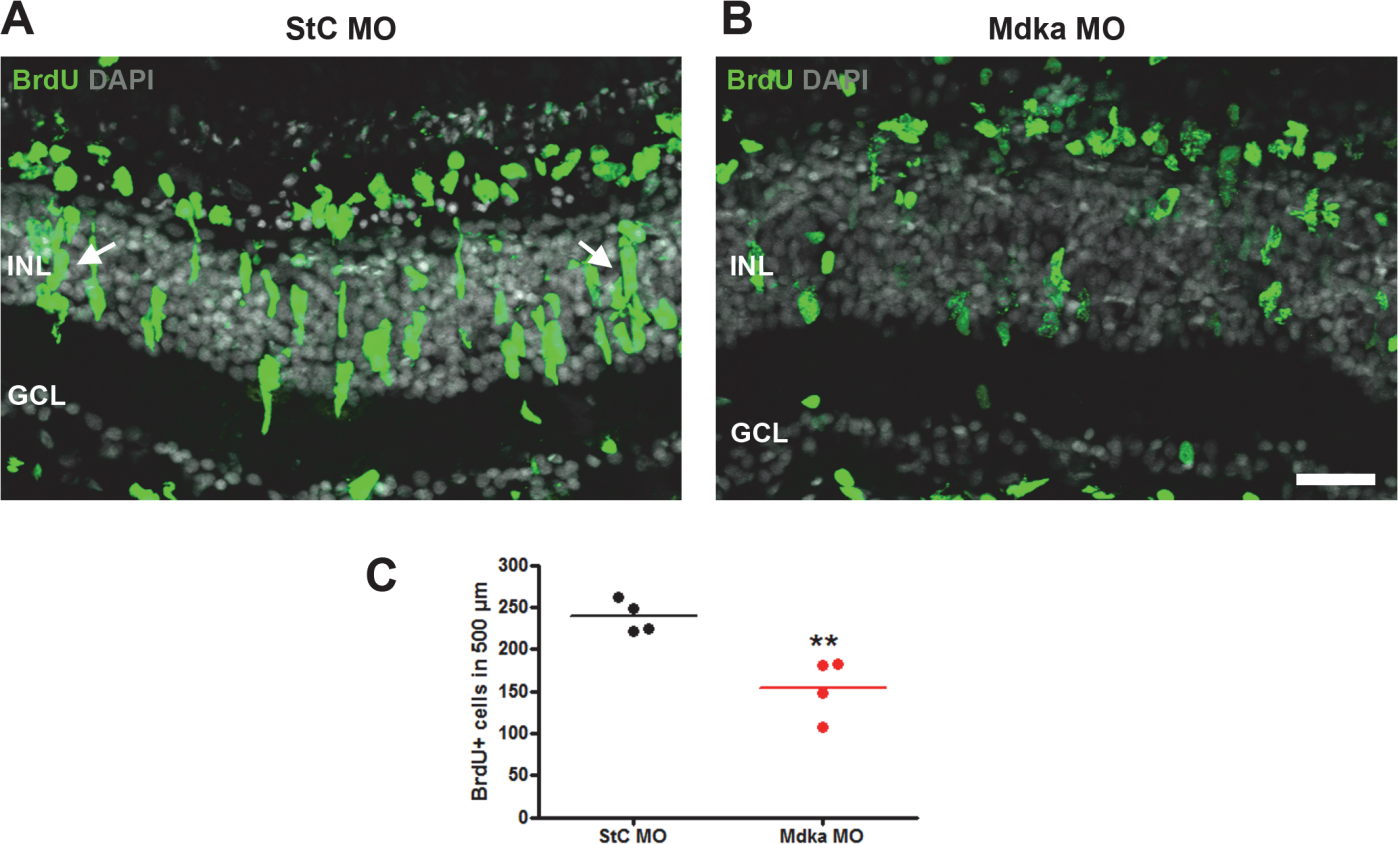
Mdka knockdown reduces the number of injury-induced progenitors. Progenitors were labeled with BrdU in the retinas of control (panel A) and experimental fish (panel B) at 3 dpl. Note the radial clusters of progenitors in the control retinas (arrows in A) and their relative absence in experimental retinas (panel B). BrdU-positive cells were counted, and this is graphically represented in panel C. INL: inner nuclear layer; GCL: ganglion cell layer; BrdU: Bromodeoxyuridine. Scale bar = 25 μm. ** p<0.01.

To test the functional role of Mdka during photoreceptor regeneration, Mdka knockdown was performed by electroporating ATG-targeted or control MOs at 1dpl (S1 Fig.). The effectiveness of the knockdown was validated by the absence of Mdka immunofluorescence in sections adjacent to those used for cell counts (S4 Fig.). In control retinas, the BrdU immunostaining reflected the well-described pattern of cell proliferation in response to photoreceptor death (e.g., [57]). Beneath the depleted photoreceptor layer, progenitors form radial clusters within the INL, surrounding each parent Müller glial stem cell, and accumulate within the nascent ONL (Fig. 5A). Following knockdown of Mdka, the average number of BrdU-positive cells per 500 μm was significantly less in underlying the lesion (239.0 ± 9.9 vs. 154.7 ± 17.7; p-value <0.01), and the proliferating cells appeared more scattered among the retinal layers (Fig. 5B,C).

**Fig 6:**
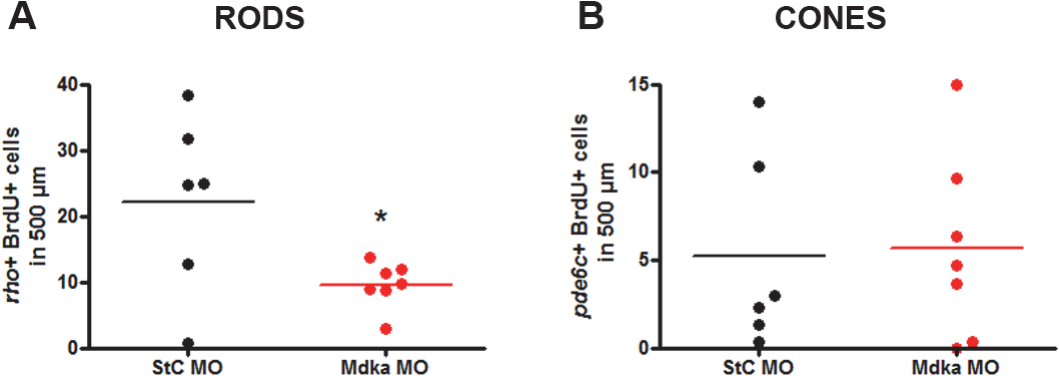
Knockdown of Mdka reduces the number of regenerated rod photoreceptors. Graphs in panels A and B represent the number of regenerated rods and cones, respectively, counted within the photolytic lesions of experimental and control fish. Bromodeoxyuridine; *rho*: *rhodopsin*; *pde6c*: *phosphodiesterase6c*. *p-value < 0.05.

The effect of Mdka knockdown on the injury-induced proliferation led us to hypothesize that Mdka may also impact the regeneration of rod and/or cone photoreceptors. For retinas at 6 dpl, *in situ* hybridizations specific for rod or cone genes were used to separately label these cells, and regenerated rods and cones were quantified. BrdU immunostaining was combined with the *in situ* labels to exclude photoreceptors that may have survived the photolytic lesions and to insure that only regenerated rods and cones were counted. Mdka knockdown was confirmed by immunostaining sample sections from each eye (data not shown). Compared to control retinas, Mdka knockdown resulted in statistically less regenerated rods in experimental retinas than controls (22.17 ± 5.5 vs. 9.631 ± 1.3; Fig. 6A). In contrast, no significant differences were found in the number of regenerated cones (Fig. 6B).

## Discussion

Developmental neurogenesis in the zebrafish retina is amazingly rapid [58], and, as in all vertebrates, is controlled by complex signaling events [59]. The zebrafish retina is formed from a pool of mitotic progenitors that begin exiting the cell cycle around 28–32 hpf [60,61] and thereafter continue to exit the cell cycle in a complex spatiotemporal pattern [62]. The cellular expression of *mdka* reflects this pattern of neurogenesis. *mdka* is expressed in all mitotic progenitors and downregulated as cells exit the cell cycle [43]. Upon retinal differentiation, *mdka* is then expressed in a single type of mature neuron, the horizontal cell, which resides in a monolayer between the inner retinal neurons, the outer plexiform layer and photoreceptors. Interestingly, the expression of *mdka* in horizontal cells is strongly regulated by the circadian rhythm [49]. Loss- and gain-of-function studies demonstrated that during retinal neurogenesis, Mdka governs cell cycle kinetics [44], perhaps, functioning in an autocrine manner. In the present study, we use immunofluorescence with polyclonal antibodies specific for Mdka to document immunolocalization in both the developing and adult retinas and during photoreceptor regeneration. In the developing retina, cellular pattern of Mdka immunostaining varies with developmental stage, reflecting the dynamic spatiotemporal pattern of the gene expression. Antibodies against Mdka stain progenitors in the embryonic retina and horizontal cells in adults. However, in both embryonic and adult retinas, the immunostaining only partially overlaps with cellular expression of *mdka*, leading us to infer that Mdka is secreted and may have paracrine functions as well. Finally, we also tested the function of Mdka during photoreceptor regeneration, and these experiments showed that Mdka governs injury-induced cell proliferation and this leads to a deficit in the regeneration of rod photoreceptors.

The mismatch in the cellular pattern of immunostaining and gene expression suggests that Mdka may play multiple roles. In the early neuroepithelium, between 24 and 36hpf, the Mdka immunostaining labels each retinal progenitor, but the densest staining is at the basal surface of the retina. Apicobasal polarity of growth factors in the retinal neuroepithelium is a well-documented mechanism for governing interkinetic nuclear migration, cell proliferation and cell fate determination [63,64]. Following knockdown of Mdka, retinal progenitors undergo interkinetic nuclear migration, but their transit from basal to apical surface is slowed and the proportion of cells that enter mitosis is diminished [44]. We speculate that the accumulation of Mdka at the basal surface of the retinal neuroepithelium contributes to apicobasal polarity and functions to govern phases of the cell cycle among progenitors in proximity to the basal surface (see also [65]). At 48hpf, when rapid cellular differentiation is happening, Mdka immunostaining at the basal surface is lost, and the immunolabel transiently accumulates in the nascent inner plexiform layer. The immunostaining of this synaptic layer is the first evidence that Mdka may be secreted and trafficked to retinal compartments separate from the *mdka-*expressing cells. In the central nervous system, Mdka plays a role promoting neurite outgrowth (reviewed in [15]; see also, [66]). Similarly, the apparently transient localization of Mdka in the developing inner plexiform layer may play a role in directing the outgrowth of immature dendritic processes and/or establishing the initial synaptic contacts made there.

The early, spatially dynamic pattern of Mdka immunostaining stabilizes as the cellular differentiation of the retina becomes complete. At and after 120 hpf, the Mdka antibodies label horizontal cells, corresponding to the unique cellular pattern of *mdka* expression. The Mdka immunolabel in horizontal cells is initially diffuse within the cytoplasm, but in adults, Mdka immunostaining is consolidated to a small plaque, which precisely colocalizes with markers of the Golgi complex. The Mdka antibodies also label rod photoreceptors in the outer nuclear layer. Numerous *in situ* hybridizations over several years and multiple investigators [43,44] indicate that rods do not express *mdka*. The colocalization of Mdka immunostaining with the Golgi complex of horizontal cells and the immunostaining of rods suggests that Mdka is synthesized by the horizontal cells, secreted in a circadian manner and sequestered by overlying rod photoreceptors. We do not know the molecular mechanisms by which Mdka associates with rod photoreceptors nor the function it mediates, however, the data provided here is, to the best of our knowledge, the first evidence of a bona fide growth factor released by horizontal cells into the outer nuclear layer.

In the adult retina, the normal pattern of Mdka immunostaining changes dramatically following the death of the photoreceptors (see also [43]). Mdka immunostaining in the horizontal cells beneath the depleted ONL is lost, and Müller glia and their basal endfeet are strongly labeled. Mdka antibodies also label the injury-induced retinal progenitors. These observations, and our previous data [44], led us to hypothesize that Mdka may govern aspects of photoreceptor regeneration. Using specific morpholinos to knockdown Mdka, we tested this hypothesis. Compared to controls, following Mdka knockdown, there were less proliferating retinal progenitors. The electroporation was performed near the time point when Müller glia enter the cell cycle following the death of photoreceptors [57], and, therefore, the loss of Mdka may either limit the ability of Müller glia to enter the cell cycle or, via inheritance of the morpholinos from parent Müller glia, inhibit proliferation of the Müller glia-derived progenitors. The reduced number progenitors following the knockdown of Midkine was accompanied by the absence of the characteristic neurogenic clusters that form around each Müller glia, and the progenitors that were present were dispersed throughout the retina. Further, following Mdka knockdown, there was a deficit in the regeneration of rod photoreceptors. We interpret this to show that decreased Mdka, similar to what was observed in the developing retina [44], slows the cell cycle, leading to a reduced number of progenitors and, by extension, a deficit in the number of regenerated rod photoreceptors. This suggests that during photoreceptor regeneration, Mdka functions to govern cell cycle kinetics, similar to what was demonstrated for this protein in the developing retina [44].

The results presented here are consistent with multiple studies implicating Midkine in the repair of the mammalian nervous system (for a review see [15]). For example, Midkine promotes functional recovery after spinal cord injury in rats [67], and in *Mdk* knockout mice, the time course of recovery from cerebral ischemia or peripheral nerve injury is delayed [13,68]. Similarly, *Mdk* knockout mice show early preclinical features of Parkinson's disease [69] and a higher vulnerability to the neurotoxic effects induced by amphetamine [70]. There is a rapidly growing body of knowledge about molecular mechanisms that underlie neuronal regeneration in the zebrafish retina (reviewed in [33,71]), and our data demonstrate that Mdka and the signaling pathways it governs can be added mechanisms that govern photoreceptor genesis. The role of Mdka in this stem cell-based regeneration opens the possibility of leveraging the Midkine signaling pathways as a potential therapeutic approach to treating degenerative disease affecting tissues within the mammalian central nervous system.

## Supporting Information

S1 Fig Experimental design used to analyze photoreceptor regeneration in the adult retina. Adult zebrafish were dark-adapted for 24 h, and photolytic lesions consisted of a 30 min exposure to high-intensity light, by three days of constant bright light. At one day post-lesion (dpl), morpholinos were injected into the vitreous cavity and electroporated into the right eye of experimental and control animals. At two dpl, fish were immersed in BrdU solution for 24 hrs. Animals were then sacrificed at three dpl for quantification of BrdU+ cells, or returned to normal light conditions and sacrificed at six dpl for quantification of regenerated rod and cone photoreceptors. BrdU: Bromodeoxyuridine; *rho*: *rhodopsin*; *pde6c*: *phosphodiesterase 6c*.
(TIF)

S2 Fig Validation of Mdka antibodies. Specificity of the anti-Mdka antibodies was determined by the selective loss of Mdka immunolabeling in Western blots (panel A) and retinal sections (panel B) from morphant embryos at 48 hpf. (A) For Western blots, β-actin served as the loading control. (B) Mdka MM—embryos injected with control morpholinos; Mdka MO—embryos injected with ATG-targeted morpholinos. Scale bar = 20 μm.
(TIF)

S3 Fig Mdka antibodies label rods, but not cones. Sections were stained with antibodies against Mdka and the rod marker Zpr3 (panel A) or the red-green cones marker Zpr1 (panel B). Note the Mdka immunostaining of the rod photoreceptor nuclei and co-localizations with rod inner segments (arrows, panel A). Zpr1 labels the cell surface of red-green cones and this marker does not colocalize with the Mdka immunostaining (panel B) ONL: outer nuclear layer; INL: inner nuclear layer. Scale bar equals 10 μm.
(TIF)

S4 Fig Effective knockdown of Mdka by *mdka* morpholinos at 6 dpl. Sections from retinas electroporated with control morpholinos show robust Mdka immunostaining (column A). In contrast, retinas electroporated with ATG-targeted morpholinos show a clear knockdown of Mdka immunostaining (column B). INL: inner nuclear layer; GCL: ganglion cell layer. Scale bar equals 50μm.
(TIF)
