# Supplementary figures and images for: Midkine-a Protein Localization in the Developing and Adult Retina of the Zebrafish and Its Function During Photoreceptor Regeneration
Source: PLoS One. 2015 Mar 24;10(3):e0121789. doi: 10.1371/journal.pone.0121789 (PMC4372396; doi:10.1371/journal.pone.0121789)

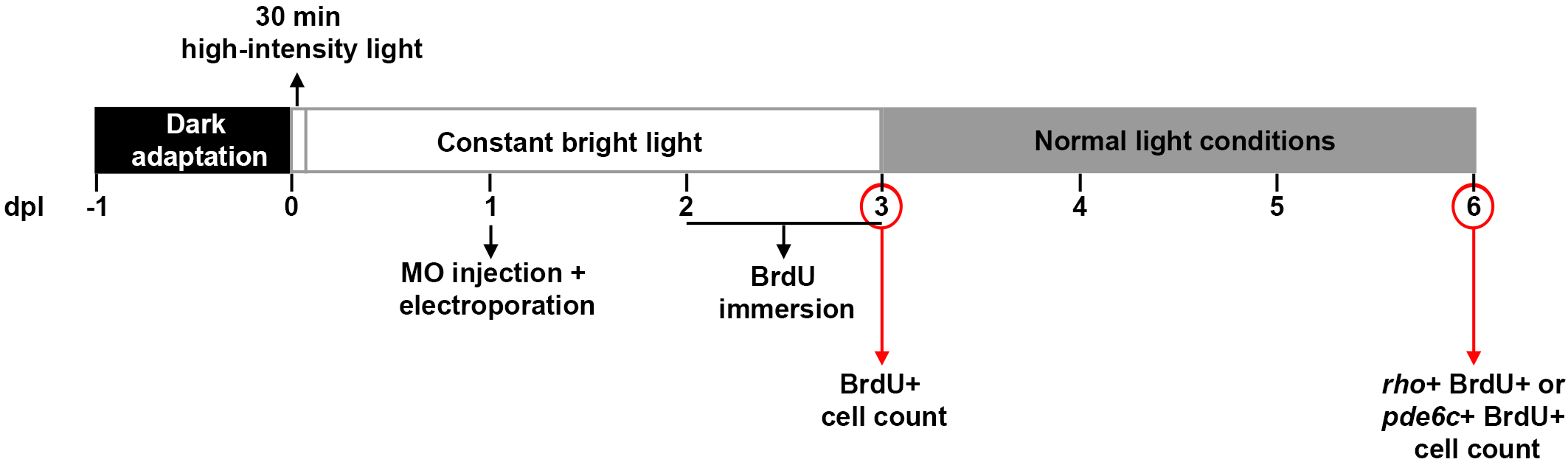

Supplement: S1 Fig — Adult zebrafish were dark-adapted for 24 h, and photolytic lesions consisted of a 30 min exposure to high-intensity light, by three days of constant bright light. At one day post-lesion (dpl), morpholinos were injected into the vitreous cavity and electroporated into the right eye of experimental and control animals. At two dpl, fish were immersed in BrdU solution for 24 hrs. Animals were then sacrificed at three dpl for quantification of BrdU+ cells, or returned to normal light conditions and sacrificed at six dpl for quantification of regenerated rod and cone photoreceptors. BrdU: Bromodeoxyuridine; rho: rhodopsin; pde6c: phosphodiesterase 6c. (TIF) [file pone.0121789.s001.tif]

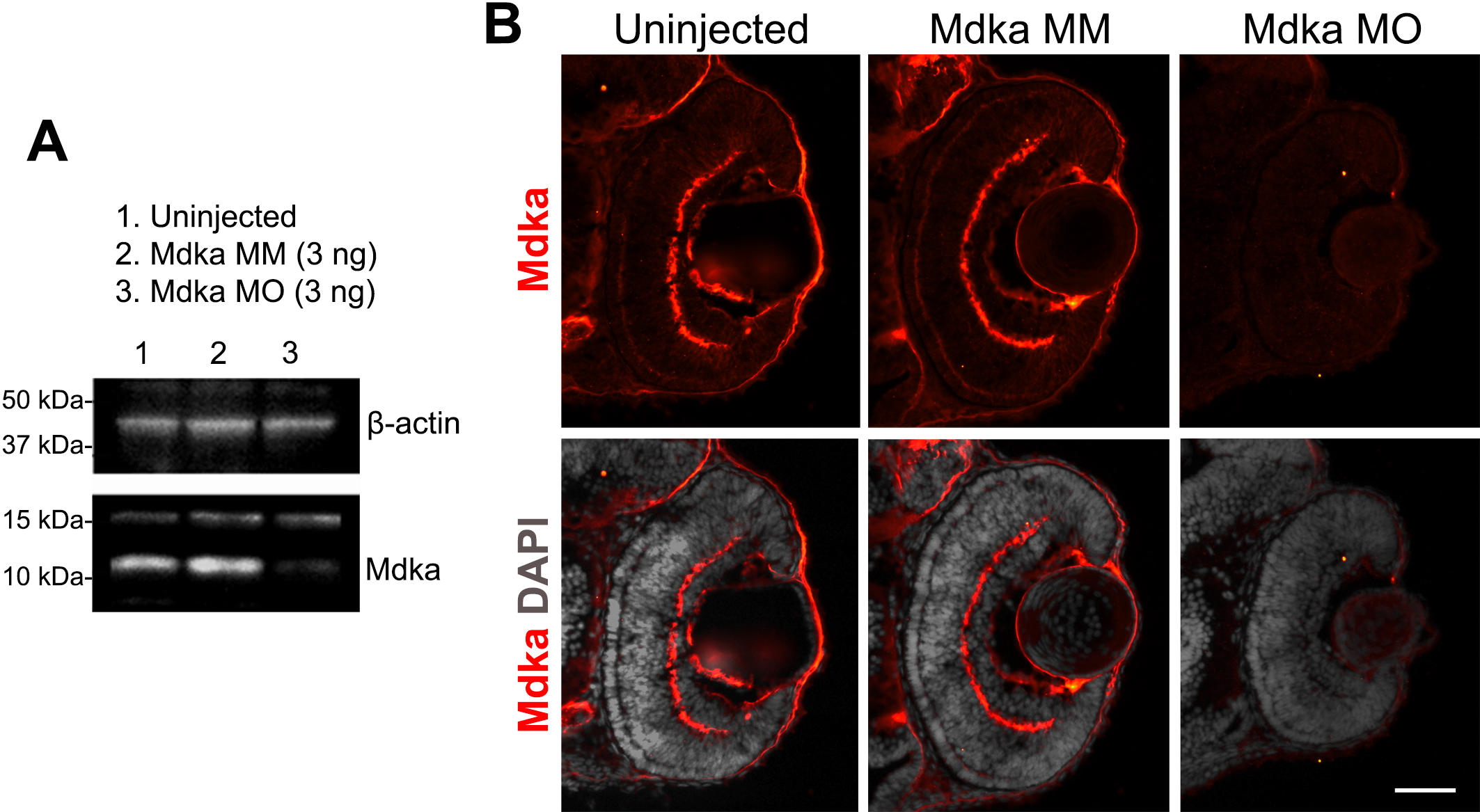

Supplement: S2 Fig — Specificity of the anti-Mdka antibodies was determined by the selective loss of Mdka immunolabeling in Western blots (panel A) and retinal sections (panel B) from morphant embryos at 48 hpf. (A) For Western blots, β-actin served as the loading control. (B) Mdka MM—embryos injected with control morpholinos; Mdka MO—embryos injected with ATG-targeted morpholinos. Scale bar = 20 μm. (TIF) [file pone.0121789.s002.tif]

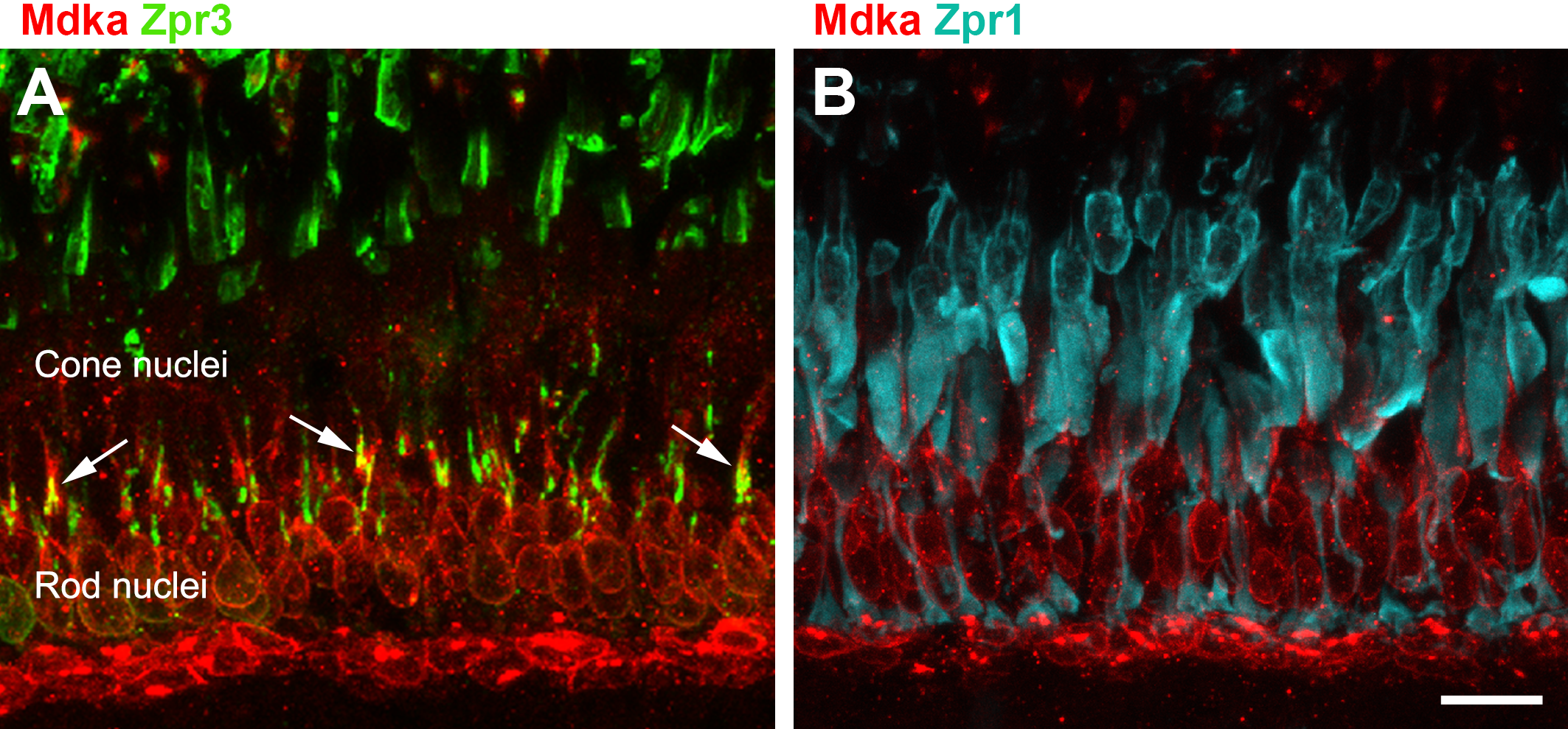

Supplement: S3 Fig — Sections were stained with antibodies against Mdka and the rod marker Zpr3 (panel A) or the red-green cones marker Zpr1 (panel B). Note the Mdka immunostaining of the rod photoreceptor nuclei and co-localizations with rod inner segments (arrows, panel A). Zpr1 labels the cell surface of red-green cones and this marker does not colocalize with the Mdka immunostaining (panel B) ONL: outer nuclear layer; INL: inner nuclear layer. Scale bar equals 10 μm. (TIF) [file pone.0121789.s003.tif]

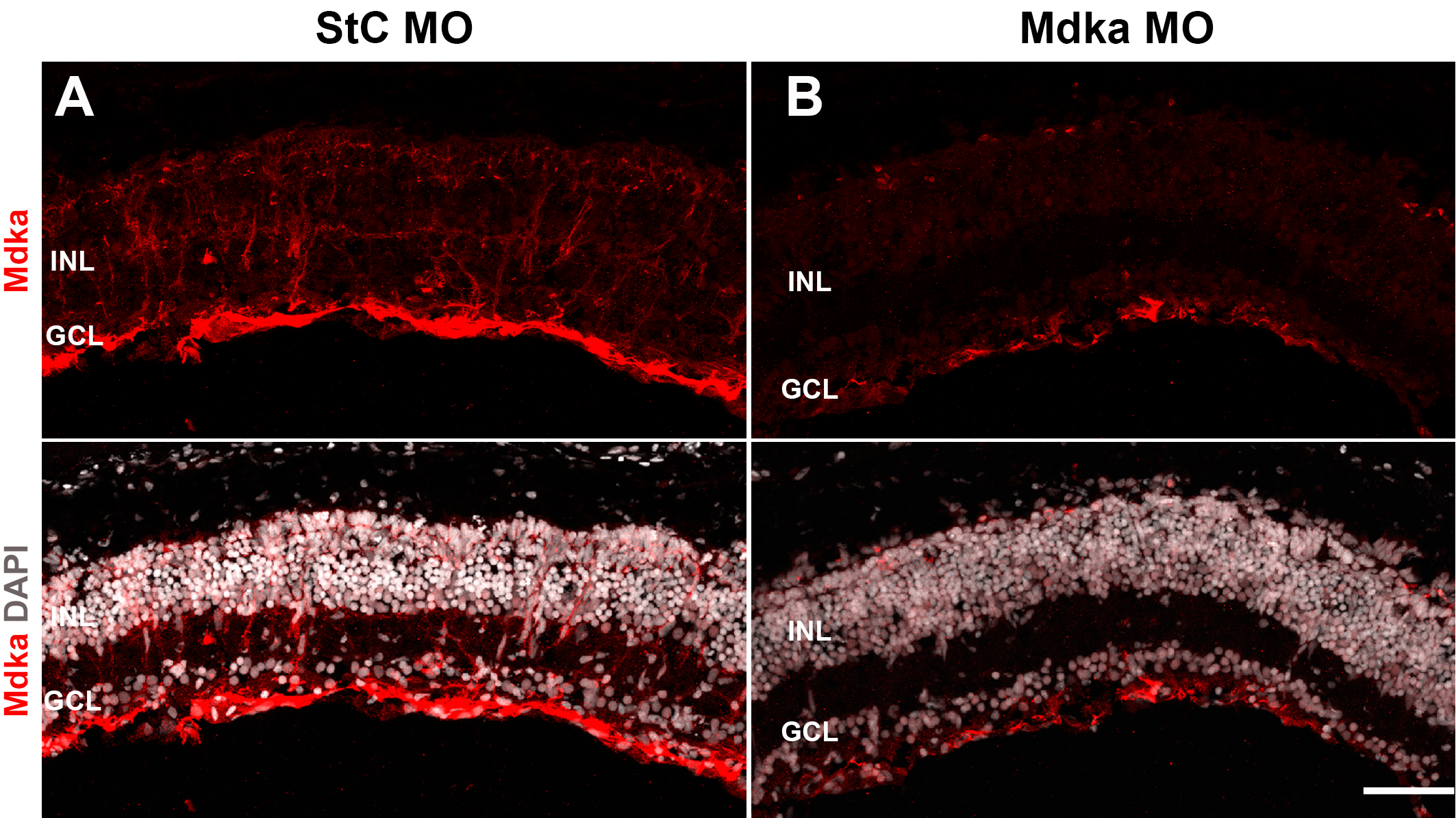

Supplement: S4 Fig — Sections from retinas electroporated with control morpholinos show robust Mdka immunostaining (column A). In contrast, retinas electroporated with ATG-targeted morpholinos show a clear knockdown of Mdka immunostaining (column B). INL: inner nuclear layer; GCL: ganglion cell layer. Scale bar equals 50μm. (TIF) [file pone.0121789.s004.tif]
